# Screening for markers of frailty and perceived risk of adverse outcomes using the Risk Instrument for Screening in the Community (RISC)

**DOI:** 10.1186/1471-2318-14-104

**Published:** 2014-09-19

**Authors:** Rónán O’Caoimh, Yang Gao, Anton Svendrovski, Elizabeth Healy, Elizabeth O’Connell, Gabrielle O’Keeffe, Una Cronin, Eileen O’Herlihy, Nicola Cornally, William D Molloy

**Affiliations:** Centre for Gerontology and Rehabilitation, University College Cork, St Finbarrs Hospital, Douglas Rd, Cork City, Ireland; UZIK Consulting Inc, 86 Gerrard St E, Unit 12D, Toronto, ON M5B 2J1 Canada; Centre for Public Health Nursing, Ballincollig and Bishopstown, Co Cork, Ireland; Centre for Public Health Nursing, Mahon and Ballintemple, Cork City, Ireland; Health Service Executive of Ireland, South Lee, St Finbarrs Hospital, Douglas Rd, Cork City, Ireland; School of Nursing, University College Cork, Cork, Ireland; COLLAGE (COLLaboration on AGEing), University College Cork, Cork City and Louth Age Friendly County Initiative, Co Louth, Ireland

**Keywords:** Screening, Frailty, Risk, Adverse outcomes, Clinical frailty scale (CFS), Risk instrument for screening in the community (RISC), Public health nurses (PHNs), Comorbidities, Cognitive impairment, Barthel index (BI), Abbreviated mental test score (AMTS)

## Abstract

**Background:**

Functional decline and frailty are common in community dwelling older adults, increasing the risk of adverse outcomes. Given this, we investigated the prevalence of frailty-associated risk factors and their distribution according to the severity of perceived risk in a cohort of community dwelling older adults, using the Risk Instrument for Screening in the Community (RISC).

**Methods:**

A cohort of 803 community dwelling older adults were scored for frailty by their public health nurse (PHN) using the Clinical Frailty Scale (CFS) and for risk of three adverse outcomes: i) institutionalisation, ii) hospitalisation and iii) death, within the next year, from one (lowest) to five (highest) using the RISC. Prior to scoring, PHNs stated whether they regarded patients as frail.

**Results:**

The median age of patients was 80 years (interquartile range 10), of whom 64% were female and 47.4% were living alone. The median Abbreviated Mental Test Score (AMTS) was 10 (0) and Barthel Index was 18/20 (6). PHNs regarded 42% of patients as frail, while the CFS categorized 54% (scoring ≥5) as frail. Dividing patients into low-risk (score one or two), medium-risk (score three) and high-risk (score four or five) using the RISC showed that 4.3% were considered high risk of institutionalization, 14.5% for hospitalization, and 2.7% for death, within one year of the assessment. There were significant differences in median CFS (4/9 versus 6/9 versus 6/9, p < 0.001), Barthel Index (18/20 versus 11/20 versus 14/20, p < 0.001) and mean AMTS scores (9.51 versus 7.57 versus 7.00, p < 0.001) between those considered low, medium and high risk of institutionalisation respectively. Differences were also statistically significant for hospitalisation and death. Age, gender and living alone were inconsistently associated with perceived risk. Frailty most closely correlated with functional impairment, r = −0.80, p < 0.001.

**Conclusion:**

The majority of patients in this community sample were perceived to be low risk for adverse outcomes. Frailty, cognitive impairment and functional status were markers of perceived risk. Age, gender and social isolation were not and may not be useful indicators when triaging community dwellers. The RISC now requires validation against adverse outcomes.

## Background

Functional decline and frailty are common in community dwelling older adults [1–3] and influence the risk of adverse outcomes. Identifying those likely to develop adverse outcomes is important in order to target limited healthcare resources in an efficient and effective manner. Risk assessment utilizing different risk prediction models is increasingly being used in the community [4]. Risk describes the amount of potential harm that can occur in a set period of time due to a specific event, or series of events and is the product of the probability that harm will occur and the magnitude of its severity [5–7]. Rational decision-making in healthcare requires reliable and valid quantitative ways of expressing risk that balance the potential costs and benefits of different management strategies [8].

Multiple factors including cognitive impairment, depression, medical comorbidities, low levels of physical activity and social isolation are associated with an increased risk of adverse outcomes [9–11]. Many but not all of these predispose to the development of frailty [12]. These factors can be grouped into three main categories or domains: mental state, activities of daily living (ADLs) and medical state. The ability of each individual’s caregiver network and social supports to manage the person’s care deficit also affects their level of risk. Inadequate social or caregiver networks predict mortality and contribute to other poor healthcare outcomes [13, 14], including institutionalization [15, 16].

A variety of different methods have been used in an attempt to identify community dwellers at risk of adverse outcomes. Many focus on the identification of frailty [17], acting as short surrogates for Comprehensive Geriatric Assessment [18]. They include direct (home assessment) [19] and indirect (postal survey) [20–22], targeted and non-targeted assessment strategies [23]. Indirect and non-targeted community screening is less efficient, suggesting that rapid screening, followed by triage and appropriate management of high-risk individuals is most effective [23]. Although the stratification of risk scores using these instruments is associated with clinically meaningful gradients of adverse outcomes [4], most risk prediction models have poor predictive ability [4]. This is especially true at an individual level and may relate to a failure to incorporate important personalized social and demographic data [4].

Community healthcare nurses known also as public health nurses (PHNs), visit patients in their home and may be in the best position to screen older people, both opportunistically and proactively. PHNs play a key role in all areas of healthcare delivery in the community, including assessment of care needs [24] and can be trained to deliver specific interventions from psychosocial strategies [25] to interventions for chronic medical conditions [26] in the home environment. In some countries, people with chronic illnesses such as dementia are more likely to be attended by PHNs than other healthcare professionals [27]. Studies have found high levels of frailty related risk factors among patients under PHN follow-up in the community [28]. Despite this, few studies have examined the role of PHNs in the care of frail and functionally impaired community dwelling older adults. In particular, few studies consider the factors that influence PHNs’ decision-making or that contribute to their interpretation of risk.

The purpose of this study was first to establish the prevalence of risk factors for frailty and functional decline in a sample of community dwelling older adults monitored by PHNs in Ireland, second to identify factors associated with perceived risk of adverse outcomes and third to investigate their distribution according to the severity of that perceived risk using a new risk stratification model, the Risk Instrument for Screening in the Community (RISC).

## Methods

### Patients

A random sample of older adults, under regular follow-up, in two PHN sectors in Cork City and County in Southern Ireland were selected by convenience sampling. Community dwellers, including those living in supervised (sheltered) accommodation, aged over 65 years and followed-up by a PHN were included. Subjects were excluded if they were in institutional care (nursing home or other long-term care unit) or under 65 years.

### Data collection and sampling

This paper reports initial findings of the Community Assessment of Risk and treatment Strategies (CARTS) study, an ongoing prospective cohort study of community dwelling older adults followed by PHNs in Southern Ireland as part of Irelands European Innovation Partnership on Active and Healthy Ageing reference site COLLAGE (COLLaboration on AGEing) [29, 30]. PHN sectors covering urban, suburban and some rural areas in southern Ireland, were approached and invited to participate. Ballincollig and Bishopstown, and Mahon and Ballintemple public health centres in County Cork were the first respondents and were sampled in that order. Sampling was thus based on the non-probability method of convenience sampling using a quota method. All PHNs (n = 15) from these centres were asked to participate and include all patients under their care who were under regular follow-up. Prior to assessing their cases, PHN’s were trained and certified in scoring the RISC (previously called the Community Assessment of Risk Screening Tool) [31]. All subjects were then scored by PHNs using this instrument. Scoring was based on the PHNs knowledge of the patients and each PHN only scored subjects directly under their care. Prior to scoring, PHNs stated whether, in their own opinion, patients were frail or not, yes or no, the PHNs’ own assessment of frailty. Additional patient information was abstracted for the PHN nursing records by a clinician, blinded to the RISC scores. This included (where available) age, gender, education, medications, medical comorbidities, indication for referral, source of referral (professional and self-referral), use of home supports and objective measures of cognition and ADL function. Ethical approval was granted by the Clinical Research Ethics Committee of the Cork Teaching Hospitals in advance of the study. Although consent was not required for retrospective chart review, informed written consent was obtained from all patients included in the CARTS intervention study. Assent was obtained from relatives of those unable to provide consent.

### Outcome measures

**Figure 1:**
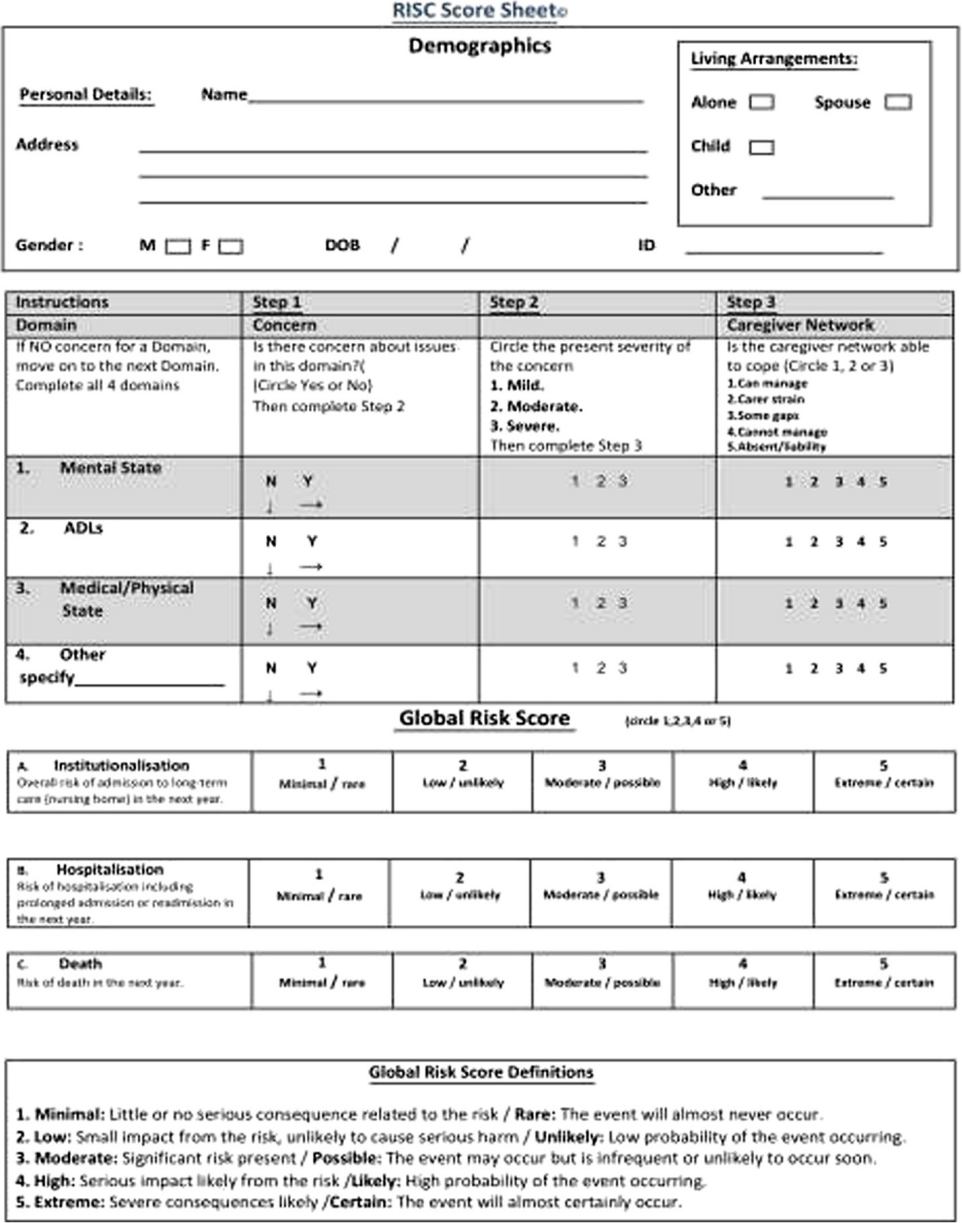
**Risk Instrument for Screening in the Community (RISC) score sheet.**

Perceived risk of three adverse outcomes namely, institutionalisation, hospitalisation and death, occurring in the next year, were scored using the RISC. Institutionalisation was defined as admission to a nursing home or other long-term care institution. Hospitalisation was defined as an acute, non-elective admission to hospital. The RISC is a new, reliable screening and assessment instrument designed to measure one-year risk of three adverse outcomes: institutionalisation, hospitalisation and death [31]. The instrument collects demographic data and records the presence and magnitude (mild, moderate, severe) of concern across three domains: mental state, ADLs and medical state. Based upon the caregiver networks’ ability to manage each domain, an overall *global risk* score is then attributed to each adverse outcome. The *global risk* score is scored using a five-point Likert scale, ranking risk from one (minimal and rare) to five (extreme and certain). The RISC consists of *Yes* or *No* responses to the presence of a concern for the three domains. Developed in conjunction with PHNs, through an iterative process, the RISC takes 2–5 minutes to complete and is currently being validated in different samples of community dwelling older adults. The RISC score sheet is presented in Figure 1.

Standardized testing, including the Barthel Index (BI) [32] and Abbreviated Mental Test Score (AMTS) [33], were conducted routinely by PHNs. The BI is a 20-point measure of basic ADL’s where a score of 20 indicates independence and 0 denotes complete functional dependence. The AMTS is a ten-point score of cognition where ten suggests normal cognition and zero severe cognitive impairment. A cut-off of seven (<7) is considered suggestive of cognitive impairment. In addition, the PHN scored each patient on the Clinical Frailty Scale (CFS) [34], a validated measure of frailty. This is a nine-point scale, scored from one (very fit) to nine (terminally ill) and can be corrected for people with dementia. Medication use and medical issues were also identified. Co-morbidity data were extracted from the PHN nursing records. The overall burden of co-morbidity was measured using the Charlson Co-morbidity Index [35].

### Statistical analysis

Data were entered into SPSS version 18.0. Kolmogorov-Smirnov and Shapiro-Wilk tests were used to test for normality. Normally distributed data were compared using Students t-tests. Non-normally distributed data were compared with the Mann–Whitney U test. Correlations were determined using Pearson’s or Spearman’s rho (non-parametric data). Kruskal-Wallis and Pearsons Chi Squared tests (categorical outcomes) were used to compare distributions.

## Results

### Demographics

The baseline demographic data is summarized in Table 1. In total 803 patients were reviewed. The most common indication for PHN involvement was review post discharge from hospital (n = 329, 41%) followed by assessment for services, including provision of aids and appliances (n = 265, 33%), wound care (n = 88, 11%) and other (n = 121, 15%). The mean age of patients, across the two sectors, was 79.8 years, with a standard deviation (SD) of +/−7.4, median age of 80 years and interquartile range (IQR) of 10. There were 516 females, (64%) and 287 males, (36%). The mean age of females was 80.2 years (+/−7.4) compared to 79 (+/−7.5) for males. Females were significantly older, (p = 0.04). Educational level was available for 415 subjects. Of these, 154 (31%) had primary education, 178 (43%) secondary and 83 (20%), third level. Overall, 374 (47.4%) subjects were living alone, most of whom were female, (n = 268, 71.7%). The sample was predominantly urban/suburban (n = 746, 93%) and the majority (n = 724, 90%) lived in their own residences. The prevalence of co-morbidities found in the study population is presented in Table 2. The median Charleston Co-morbidity Index score was one (two) and scores ranged from 0–10. The mean number of total prescription medications prescribed per person was 5.5 (+/−3.4), median of five (5), range 0–18.

**Table 1:** **Baseline demographic data including a comparison between those scoring minimum (score 1 and 2/5) and maximum (score 3, 4 and 5/5) risk on the Risk Instrument for Screening in the Community (RISC)**

Descriptor	Total (Percentage)	RISC institutionalization		P-value	RISC hospitalization		P-value	RISC death		P-value
		Minimum risk	Maximum risk		Minimum risk	Maximum risk		Maximum risk	Maximum risk	
		N = 686	N = 96		N = 499	N = 283		N = 621	N = 161	
Age (Mean ± SD)	79.8 ± 7.4	79.7 ± 7.4	81.8 ± 6.7	< 0.01	80 ± 7.3	79.9 ± 7.4	0.99	79 ± 7	83.2 ± 7.7	< 0.001
Gender	F 516 (64%)	65%	67%	0.70	69%	57%	0.001	67%	59%	0.08
	M 287 (36%)	35%	33%		31%	43%		33%	41%	
Educational Level (where available)				0.02			0.06			< 0.01
- Primary	154 (37%)	35%	48%		33%	42%		33%	50%	
- Secondary	178 (43%)	46%	27%		48%	36%		47%	30%	
- Third	83 (20%)	19%	25%		19%	22%		20%	20%	
Living alone	Y 381 (47%)	48%	41%	0.18	52%	40%	< 0.01	49%	43%	0.20
	N 422 (53%)	52%	59%		48%	60%		51%	57%	
Accommodation type										0.22
- Own home	724 (90%)	93%	85%	< 0.01	93%	89%	0.02	93%	89%	
- Sheltered accommodation	21 (3%)	1%	0%		0%	2%		1%	1%	
- Another’s home	58 (7%)	6%	15%		6%	9%		6%	10%	
Home Help	Y 419 (52%)	50%	81%	< 0.001	48%	63%	< 0.001	53%	57%	0.29
(Any type)	N 384 (48%)	50%	19%		52%	37%		47%	43%	
Home help hours										< .001
0	384 (48%)	58%	21%	< 0.001	60%	42%	< 0.001	55%	49%	
≤5	335 (41%)	34%	44%		34%	39%		37%	31%	
6-10	40 (5%)	4%	14%		4%	8%		5%	7%	
11-20	24 (3%)	2%	10%		2%	5%		3%	6%	
>20	20 (3%)	2%	11%		1%	6%		1%	7%	
Abbreviated Mental Test Score (Mean ± SD)	9.25 ± 1.86	9.51 ± 1.46	7.38 ± 3.07	< 0.001	9.43 ± 1.60	8.94 ± 2.23	< 0.001	9.41 ± 1.58	8.64 ± 2.61	< 0.001
Cognitive Impairment	Y 134 (16.7%)	21%	75%	< 0.001	22%	41%	< 0.001	24%	47%	< 0.001
	N 669 (83.3%)	79%	25%		78%	59%		76%	53%	
Barthel Index Score (Median ± IQR)	18 ± 6, range 0-20	18 ± 4, 0-20	12 ± 6, 0-20	< 0.001	19 ± 4, 0-20	16 ± 7, 0-20	< 0.001	18 ± 4, 0-20	15 ± 9, 0-20	< 0.001
PHNs own opinion of frailty	Y 335 (42%)	37%	76%	< 0.001	71%	63%	< 0.001	36%	65%	< 0.001
	N 468 (58%)	63%	24%		29%	37%		64%	35%	
Clinical Frailty Scale Score (Median ± IQR)	5 ± 2, range 0-9	4 ± 3, 0-9	6 ± 1, 2-8	< 0.001	4 ± 2, 0-8	6 ± 2, 2-9	< 0.001	4 ± 3, 0-8	6 ± 2, 2-9	< 0.001
Clinical Frailty Score ≥5	422 (54%)	49%	91%	< 0.001	44%	73%	< 0.001	49%	75%	< 0.001
Charlson Comorbidity Index (Median ± IQR)	1 ± 2, range 0-10	1 ± 2, 0-9	2 ± 2, 0-10	< 0.001	1 ± 1, 0-7	2 ± 2, 0-10	< 0.001	1 ± 2, 0-8	2 ± 2, 0-10	< 0.001
Total number medications (Median ± IQR)	5 ± 5, range 0-18	5 ± 4.3, 0-18	7 ± 5, 1-16	0.001	4 ± 5, 0-14	7 ± 5, 0-18	< 0.001	5 ± 4, 0-16	6 ± 5, 0-18	< 0.001
Admissions in last year (Median ± IQR)	1 ± 1, range 0-10	1 ± 1, 0-10	1 ± 0, 1-5	0.17	1 ± 1, 0-6	1 ± 1, 1-10	> 0.05	1 ± 1, 0-10	1 ± 1, 1-6	0.19

**Table 2:** **Prevalence of documented co-morbidities abstracted from nursing records for patients under care of their Public Health Nurse (n = 803)**

Condition	Prevalence % (n = 803)
Hypertension	311 (39%)
Osteoarthritis	285 (36%)
Ischaemic heart disease	150 (19%)
Dementia	134 (16.7%)
Cancer (any history including skin)	127 (16%)
Anxiety-depression	119 (15%)
Atrial Fibrillation (any history)	98 (12%)
Diabetes mellitus (type 1 or 2)	91 (11%)
Stroke (infarction)	79 (10%)
Hypothyroidism	62 (8%)
Chronic obstructive pulmonary disease	59 (7%)
Venous hypertension	49 (6%)
Peripheral vascular disease	43 (5%)
Alcohol excess	40 (5%)
Parkinson’s disease	32 (4%)
Chronic kidney disease	27 (3%)
Psychosis (including schizophrenia, bipolar affective disorder)	25 (3%)
Congestive cardiac failure	23 (3%)
Seizure disorder	23 (3%)

### Cognition

The mean AMTS was 9.26 (+/−1.7) with a median of 10 (0). In all, 134 patients were regarded as having cognitive impairment, a cohort prevalence of 16.7%. The mean age of those with cognitive impairment was 81.8 years (+/−7), significantly older than those with normal cognition (mean 79.4, SD +/−7.5, p = 0.001). Subjects with cognitive impairment were more likely to be female, 70% versus 62.9% (p < 0.001) and more functionally impaired than those without documented cognitive impairment (median BI 14 versus 18, p < 0.001). Patients with cognitive impairment were as likely to be living alone as those without documented cognitive impairment, p = 0.11. There was a strong significant correlation between the AMTS and a documented diagnosis of cognitive impairment (r = 0.77, p < 0.001). There was a medium significant correlation between the BI and both cognitive impairment (r = −0.4, p < 0.001) and AMTS scores (r = 0.35, p < 0.001).

### Function and frailty

**Figure 2:**
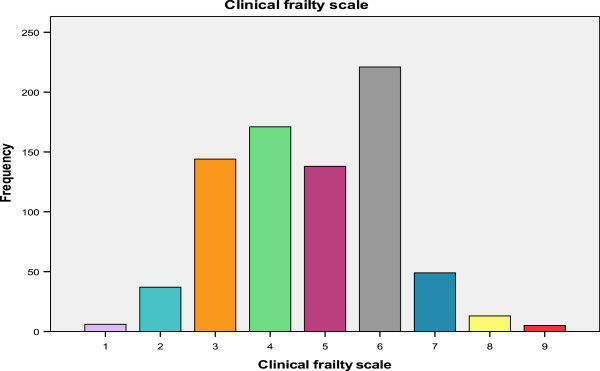
**Distribution of clinical frailty scale scores in the study population (n = 784).**

The median BI score for the total population was 18 (6). There was no statistically significant difference in BI scores between males and females, both had a median score of 18, z = −1.22, p = 0.23. There was no correlation between age and functional ability, r = −0.150, p < 0.001 using the BI score. The PHNs own opinion of frailty, without use of a standardized frailty instrument, described 335 (42%) subjects as frail. Of these, 130 (39%) were living alone. The CFS was available for 784 patients (97%), 426 (54.3%) of whom scored >5 and were categorized as *mildly frail-terminally ill*. A further 171 (21.8%) scored 4, *vulnerable*, while 187 (23.9%) scored three or less, *very fit-managing well*. The distribution of CFS scores is presented in Figure 2. The correlation between the CFS and the variables collected is presented in Table 3. There was a strong, significant correlation between the PHNs own opinion of frailty and the CFS, r = 0.56, p < 0.001, and between the BI and the CFS, r = −0.80, p < 0.001. There was small to medium, correlation between the Charleston Co-Morbidity Index and the CFS (r = 0.29, p < 0.001) and small or no correlation between the CFS and age (r = 0.12, p = 0.001), and gender (r = 0.05, p = 0.2). The CFS also correlated moderately and significantly with the RISC scores, r = 0.43 (institutionalization), r = 0.43 (hospitalization) and r = 0.35 (death), p < 0.001 (see Table 3). Comparing the PHNs own assessment of frailty to the CFS classification of frailty (score of four or less is non-frail) showed that the nurses opinion had a sensitivity of 65% and specificity of 85.7% for frailty with a positive predictive value of 84.3% and negative predicative value of 67.5%.

**Table 3:** **Correlation of patient variables to the level of frailty as measured by the Clinical Frailty Scale**

Variable	Correlation to clinical frailty scale (p value)
Age	0.12 (p = 0.001)
Gender	0.05 (p = 0.2)
Living alone	0.21 (p < 0.001)
Nurses “own” opinion of frailty	0.56 (p < 0.001)
Barthel Index	−0.80 (p < 0.001)
Cognitive impairment (documented)	0.38 (p < 0.001)
Abbreviated mental test score	0.36 (p < 0.001)
Charlson comorbidity index	0.29 (p < 0.001)
Total number of medications	0.25 (p < 0.001)
No of hospital admissions in the last year	0.19 (P < 0.001)
Receiving home help (any type)	0.32 (p < 0.001)
RISC score:	
- *global risk* institutionalisation	0.43 (p < 0.001)
- *global risk* hospitalisation	0.43 (p < 0.001)
- *global risk* death	0.35 (p < 0.001)

### Risk scores

**Figure 3:**
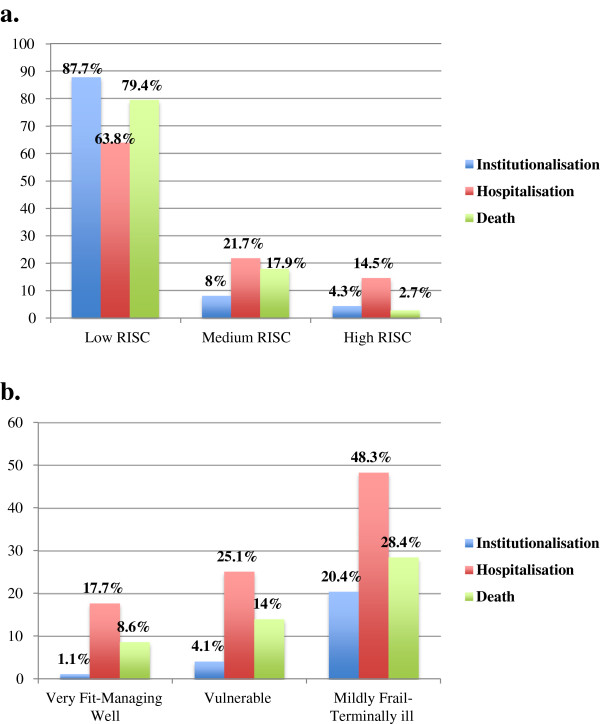
**The prevalence of perceived one-year risk of adverse outcomes of institutionalisation, hospitalisation and death, for available patients assessed using the a) Risk Instrument for Screening in the Community (RISC) and b) the prevalence of RISC outcomes according to the Clinical Frailty Scale (CFS), n = 784.**

Of those reviewed, RISC scores were available for 782 patients. After pooling the risk levels into low-risk (a score of one or two), medium-risk (score of three) and high-risk (score of four or five) using the RISC, 4.3% of the total sample were perceived by their PHN to be at high risk of institutionalisation, 14.5% at high risk of hospitalisation, and 2.7% at high risk of death, within one year of the assessment. The presence of documented cognitive impairment increased the perceived risk of institutionalisation for the high-risk group to 15%. Overall, the majority of patients were regarded as low risk for each of the adverse outcomes. This is illustrated in Figure 3a. There was a significant difference in median CFS (4/9 ± 3 versus 6/9 ± 1 versus 6/9 ± 0, p < 0.001), Barthel Index (18/20 ± 4 versus 11/20 ± 7 versus 14/20 ± 7, p < 0.001) and mean AMTS scores (9.51 ± 1.46 versus 7.57 ± 3.11 versus 7.00 ± 2.99, p < 0.001) between patients considered low, medium and high risk of institutionalisation respectively. Differences were also statistically significant for hospitalisation and death.

Further dividing patients into minimum (score of one or two, n = 686) or maximum-risk (scoring three, four or five on the RISC, n = 96) increased the percentage considered at risk to 12.3%, 36.2% and 20.6% for institutionalisation, hospitalisation and death respectively within one year of the assessment. Patients considered to be at maximum-risk of insitutionalisation were more likely to be frail as judged by the CFS (49% versus 91%, p < 0.001), functionally impaired (BI of 18/20 versus 12/20,p < 0.001) and cognitively impaired (21% versus 75%, p < 0.001). This association was similar for those considered at maximum risk of hospitalisation and death, see Table 1. Gender and living alone were not associated with perceived risk of institutionalisation or death. Age was not associated with perceived risk of hospitalisation. Most variables were significantly different for all three adverse outcomes including the prevalence of cognitive impairment (AMTS scores), functional impairment (BI scores), frailty (CFS and PHNs own opinion of frailty scores) and comorbidities (Charlson Comorbidity Index scores), see Table 1. The prevalence of RISC outcomes, using a score of three or higher as the cut-off for maximum risk, according to the CFS, is presented in Figure 3b. Patients CFS scores were divided into *very fit-managing well* (score 1–3), *vulnerable* (score of 4) and *mildly frail-terminally ill* (score 5–9), n = 784. Perceived risk of hospitalisation was greater than institutionalisation and death for all three CFS frailty levels with 48.3% of *mildly frail-terminally ill* patients perceived to be at maximum risk of hospitalisation within one year, significantly different from *very fit-managing well* (17.7%) and *vulnerable* (25.1%) patients, (χ^2^(2) = 62.37,p < 0.001). Perceived risk was also significantly different according to the frailty level for institutionalisation (χ^2^(2) = 58.17,p < 0.001) and death (χ^2^(2) = 36.65, p < 0.001).

## Discussion

This paper presents the prevalence of several established predictors of functional decline and frailty among community dwelling older adults being monitored by their PHN. It also presents the type and prevalence of factors that contribute to PHNs’ perceived risk of adverse outcomes, scored using a new screening and assessment instrument, the RISC. There was a high prevalence (54%) of frailty, as defined by CFS scores of 5 to 9 (*mildly frail-terminally ill)* [34], among this cohort of community dwellers. This was expected, given that patients being followed by PHNs are a selected sample of older adults and are more likely to have medical and other co-morbidities than a cross sectional sample of all community dwelling older adults.

Several variables correlated strongly with frailty. These included function as determined by the BI and notably PHNs own “opinion” of frailty. The close correlation of the CFS to the BI likely reflects the scoring mechanism of the CFS, which depends on the functional stage of the patient. That the opinion of PHNs in this sample correlated strongly and significantly with a validated measure of frailty, the CFS [34], suggests that healthcare workers, familiar with their patients, accurately predict risk without the need for standardized assessment instruments. Several established factors such as age [36] and female gender [37], that might increase the likelihood of frailty, were not found to correlate with the CFS. Of the other risks identified, CI in particular, correlated with the degree of functional impairment (BI). Age itself did not impact upon function or frailty. However, several challenges remain in separating frailty as a concept from the individual factors that are associated with it. It is not established whether these factors, including markers of cognition and functional impairment, cause or merely reflect the development of frailty. Future studies should include established objective markers, such as those included in the Fried criteria [38] like weight, grip strength and walking speed, which may help clarify the interaction between the components of frailty and the frailty phenotype as a whole.

In this sample, only a small percentage of the total older adult population was perceived to be living at risk. Perceived risk of adverse outcomes, within one year of assessment was generally low. This corresponds with low prevalence rates of institutionalisation and death from the community. Previous analysis of a risk register of community dwelling older adults in County Cork, Ireland, found a similar prevalence of perceived risk [39]. In that sample the composite risk of all adverse outcomes was measured at 7%. In this study, cognition, functional level and frailty correlated with the perceived risk of institutionalisation, although gender and social isolation did not. In particular, cognitive impairment increased the perceived risk of all adverse outcomes, suggesting that these patients should receive particular attention when they live in the community. Pooling results, into low, medium and high or indeed into minimum and maximum increased the number of patients perceived to be at increased risk. Perceived risks were significantly greater for those rated as frail on the CFS compared with those rated as non-frail or frail. Perceived risk of hospitalisation was higher than for the other adverse outcomes. Most patients however, even in this highly selected community sample, were regarded as being low risk of adverse events.

This paper has several limitations. The data collection was based upon a retrospective review of the patients’ PHN records and the analysis depended upon these being accurate and up to date. Some demographic data including 21 RISC and 19 CFS scores were not available for patients that had not been reviewed within the last six months. Sampling PHN records may also have led to selection bias in that patients followed by their PHN are inherently at higher risk of adverse outcomes compared to the general older adult population. The method of sampling may also have created bias. However, with quota sampling investigators are less concerned with having sufficient numbers to match the proportions in the entire population, but instead aim to sample enough patients to ensure that even small subgroups are adequately represented. Additionally, it is not certain that all PHNs correctly classified patients according to their risk. The study was however, conducted in conjunction with each patient’s PHN, who know their patients well and provide care for them over several years. Another limitation is that since the RISC is still being validated, the ability to generalize and prognosticate on the significance of the risk factors associated with the perceived risk identified are reduced. Once validated, further analysis will be required to investigate the association of these markers of frailty and perceived risk with the outcome data. Likewise, the optimal cut-off point for each adverse outcome is not yet established. The CFS has also not been validated with nurses, which may have lead to bias. Although we assessed the inter-rater reliability of the RISC, the reliability of the CFS was not determined. The subjectivity of the frailty assessment is another limitation. Both the PHNs own assessment of frailty and the CFS are subjective measures, and the inclusion of an objective observer rated assessment instrument would have reduced potential bias. Furthermore, the screening tools used for cognition and function in this study are not gold standard instruments and may have under-estimated the true prevalence of cognitive and functional impairment in this population. The AMTS is less accurate than many other short cognitive screens such the Quick mild cognitive impairment screen [40] and the Montreal Cognitive Assessment [41] and is particularly insensitive at differentiating mild cognitive impairment from normal cognition and early dementia. Likewise, the BI is a crude gauge of function and does not score instrumental ADLs. These instruments are however, widely used and are the prescribed instruments in use in the community in this region. The Charleston Co-morbidity Index is criticized for its poor predictive validity, particularly among older adults [42]. In particular, it fails to incorporate medical conditions like Parkinson’s disease, multiple sclerosis and inflammatory bowel disorders, which may contribute to comorbidity.

The strengths of this paper are the comprehensive nature of the review of the PHN records, conducted in busy health centres and the inclusion of a large cross-sectional and representative community sample, increasing the generalizability of the results. The prevalence of comorbidities reported in this study are similar to other community samples of other PHNs in Ireland [28]. A retrospective cross sectional clinical audit carried out in Dublin City, Ireland, investigating the prevalence of four frailty-related risk factors identified similar rates of suspected cognitive (16.4%) and functional impairment (BI score ≤15, 23.5%) albeit they were slightly higher in this study at 16.7% and 30.6% respectively [28].

The RISC was developed to measure risk patients' levels and is in the process of being validated. It was designed with PHNs for use in a community setting and shows excellent inter-rater reliability (IRR) [31]. This research is ongoing, and a follow-up is underway to ascertain the current status of the patients and evaluate the predictive validity of the RISC to these adverse outcomes in this population. This prospective cohort study will investigate if risk scores, described by the RISC, predict these adverse outcomes. Targeting limited resources, to medium and high-risk individuals (maximum risk), could improve efficiency in the use of limited healthcare resources [43]. Future studies will investigate if the RISC, aligned to tailored intervention programmes or care bundles, can reduce risk and incidence of adverse outcomes in community dwelling older adults.

## Conclusions

In this study the majority of community dwelling older adults were perceived by their PHN to be at minimum risk of adverse outcomes. This may facilitate targeting of these patients to prevent or postpone adverse outcomes. On the other hand, there was a large proportion of frail older adults (54%; ≥5 on the CFS) found in this study, and it remains to be seen if simple, albeit multidimensional, risk scores like the RISC may be more efficient in targeting patients for Comprehensive Geriatric Assessment than instruments like the CFS. Frailty (subjective or objective), cognitive impairment and functional status were markers of perceived risk. PHNs opinion appears to correlate with a validated frailty scale, supporting the utility of nurses in triaging patients in the community. Several factors traditionally associated with frailty such as age, gender and social isolation did not correlate with the CFS, RISC or with PHNs’ perceived risk of adverse outcomes, suggesting that despite their high prevalence, they may not be useful indicators for triaging community dwellers.
